# The impact of briefly observing faces in opaque facial masks on emotion recognition and empathic concern

**DOI:** 10.1177/17470218221092590

**Published:** 2022-04-27

**Authors:** Josh Liam Shepherd, Daniel Rippon

**Affiliations:** Faculty of Health and Life Sciences, Northumbria University, Newcastle Upon Tyne, UK

**Keywords:** Emotion recognition, facial expression, non-verbal communication, facial emotion recognition, face masks

## Abstract

Since the outbreak of SARS-CoV-2 in 2019, there have been global public health initiatives that have advocated for the community use of face masks to reduce spread of the virus. Although the community use of facial coverings has been deemed essential for public health, there have been calls for enquiries to ascertain how face masks may impact non-verbal methods of communication. This study aimed to ascertain how the brief observations of faces in opaque facial coverings could impact facial emotion recognition. It was also an aim to ascertain if there was an association between the levels of empathic concern and facial emotion recognition when viewing masked faces. An opportunity sample of 199 participants, who resided in the United Kingdom, were randomly assigned to briefly observe either masked (*n* = 102) or unmasked (*n* = 97) faces. Participants in both conditions were required to view a series of facial expressions, from the Radboud Faces Database, with models conveying the emotional states of anger, disgust, fear, happiness, sadness, and surprised. Each face was presented to participants for a period of 250 ms in the masked and unmasked conditions. A 6 (emotion type) x 2 (masked/unmasked condition) mixed ANOVA revealed that viewing masked faces significantly reduced facial emotion recognition of disgust, fear, happiness, sadness, and surprised. However, there were no differences in the success rate of recognising the emotional state of anger between the masked and unmasked conditions. Furthermore, higher levels of empathic concern were associated with greater success in facially recognising the emotional state of disgust. The results of this study suggest that significant reductions in emotion recognition, when viewing faces in opaque masks, can still be observed when people are exposed to facial stimuli for a brief period of time.

## Introduction

In 2020, the World Health Organisation (2020) published guidelines on the recommended community use of face masks as part of a public health strategy to limit the spread of SARS-CoV-2. The correct application of surgical masks, N95 masks, and cotton masks, covering the mouth and nose, have shown to be effective in providing some protection against droplet and airborne spread of SARS-CoV-2 (Ueki et al., 2020). Furthermore, widespread community use of face masks in public settings can be effective in reducing the spread of SARS-CoV-2 (Lyu & Wehby, 2020). Thus, the essential requirement to implement public health strategies that aim to mitigate the spread of SARS-CoV-2 has seen an increased use of face masks in public settings. Although facial coverings have been deemed essential for public health, there has been some enquiry on how the use of opaque face masks could impact non-verbal means of communication in community settings due to their occlusion of face regions that contribute to the holistic facial expression of emotions (Campagne, 2021). Therefore, the observation of facial expressions that convey particular emotional states can be an important facet in ensuring cohesive interpersonal communications and social interactions (Phutela, 2015). Previous studies, which have investigated how face masks may affect the ability to facially recognise emotional states, typically present participants with facial stimuli in which the mouth and nose areas are occluded but the eye region remains unobscured (Carbon, 2020; Grundmann et al., 2021). It has been posited that people may still be able to recognise mental states in others through observation of the eye region alone, which is often unobscured when wearing surgical-type face masks (Carragher & Hancock, 2020). The Reading the Mind in the Eyes test (Baron-Cohen et al., 2001) was developed to assess peoples’ ability to correctly assign mental states and feelings to others through the sole observation of the eye region. The Reading the Mind in the Eyes test has been used to illustrate that people can identify complex mental states in others, through observation of the eye region alone, with a level of accuracy that is greater than chance (Schmidtmann et al., 2020). This would suggest that people may be able to facially recognise emotions in others when viewing faces in which facial coverings leave the eye region unobscured. Therefore, there is a need to address the uncertainty concerning the extent to which observing faces in opaque facial masks may influence the ability to facially recognise the emotional states of others.

Ekman’s (1992) *Discrete Emotion Assumption* posits that humans have the capacity to non-verbally express six key universal emotional states via facial expressions; 1) happiness, 2) sadness, 3) anger, 4) disgust, 5) surprise and 6) fear (Ekman, 2004; Ekman & Friesen, 1971). Ekman and Friesen (1978) coined the term *Facial Action Coding System* when describing the process of how shifts in the muscular and structural compositions of facial features can be used to express particular emotional states in a non-verbal manner. For example, the zygomaticus major muscle, responsible for the superiorly and posteriorly contracting of the facial cheeks, has been linked with the smile expression and the communication of happiness (Beaudry et al., 2014; Dimberg & Petterson, 2000; Eisenbarth & Alpers, 2011). The medial contraction of the corrugator supercilii muscle between the eyebrows elicits a frown and has been associated with the expression of anger (Heckmann et al., 2003; Tipples et al., 2002). Typically, the upper facial features around the eyes have been associated with the presentation of sadness, fear, and anger, whereas happiness and disgust are communicated with the lower section of the face around the mouth area (Wegrzyn et al., 2017). This would suggest that facial masks that cover the lower section of the face may hinder the recognition of particular emotional states such as happiness and disgust.

There are theoretical viewpoints that could provide explanations on how the occlusion of particular areas of the face may impact the cognitive processes required for facial recognition of emotional states. Maurer et al. (2002) theory on configural face processing illustrates how humans process the first-order relations of faces or the arrangement of internal facial features (for example, two eyes above the nose and a mouth below the nose). Humans also process the second-order spatial relationships between the facial features, which is an assessment of how far apart the eyes, nose, and mouth are. This enables humans to process faces in a holistic or gestalt manner so that the features and composition of the face can be interpreted as a whole for facial coding and recognition of emotional states (Farah et al., 1938; Guo, 2012; Vaidya et al., 2014; Young et al., 2013). During facial coding, humans assess the shape, colour, and depth of individual features to recognise particular emotional states (Drummond, 2017). For example, lowered eyebrows can be recognised as the presentation of anger (Wegrzyn et al., 2017). It has been posited that any impediment to first order, second order or holistic processing of faces could inhibit the process of facial emotion recognition (Bombari et al., 2013; Calder et al., 2000). Thus, masks may cover features of the face that are necessary for the cognitive processes that underpin facial coding and recognition of emotional states.

An evolutionary perspective has posited that the ability to facially recognise emotional states can elicit adaptive behavioural responses within the observer to ensure survival and avoidance of endangerment (Ekman, 1997). For example, the ability to facially recognise the emotion of anger could elicit a prompt behavioural response of disengaging from or evading potentially aggressive acts from others (Fox et al., 2002). It has been observed that different emotional expressions can determine the type of behavioural response exhibited by observers. For example, the facial recognition of anger may prompt an avoidant response, whereas recognising the facial expression of fear can elicit observers to approach or help others who present as being fearful (Marsh et al., 2005). This is salient with Van Kleef’s (2009) Emotion as Social Information Model (EASI), which posits that facial expressions can be used as non-verbal signals of emotional states that can provide information to and influence the behavioural responses of observers. Thus, the ability to recognise facial emotions can be integral in ensuring cohesive social interactions with others within various settings. For example, within settings such as banks, it has been observed that employees who facially express a smile can elicit positive affect within customers and enhance the customer service experience (Pugh, 2001). Recognising facial expressions of emotion has also been identified as a key communication skill for frontline health care staff in developing and maintaining therapeutic relationships with patients (Roter et al., 2006). However, in response to the COVID-19 pandemic, there have been public health strategies that have mandated the communal use of masks within various indoor settings, such as in hospitality, health care services, and public transport (Allison et al., 2021). Thus, given that, the ability to recognise the emotional states of others can be important in eliciting appropriate behavioural responses that may ensure safety and also facilitate cohesive social interactions, it is necessary to ascertain how masks may influence the accuracy in facially recognising emotions.

There have been some notable investigations on how the use of facial coverings may influence people’s ability to recognise the emotional states of others. Research conducted by Carbon (2020) utilised the MPI FACE battery to illustrate how the application of surgical masks to facial stimuli can significantly reduce the recognition of anger, disgust, happiness, and sadness. Furthermore, it was observed that viewing masked faces could lead to misinterpretations of emotional states. For example, the emotion of disgust was observed to be misidentified as anger. These findings converge with Grundmann et al. (2021) who also observed that presenting faces in surgical masks can significantly reduce facial emotion recognition. However, within the study conducted by Carbon (2020), participants were not provided a stipulated time limit when viewing the masked and unmasked facial stimuli. In the study conducted by Grundmann et al. (2021), participants viewed the facial stimuli for a 2s period. Given the recent increase in the global use of face masks to reduce transmission of SARS-CoV-2 (World Health Organisation, 2020), it could be argued that the number of brief interactions or observations of masked faces within community settings have also increased. The process of facial coding and correctly recognising an emotional state can occur rapidly within 120–170 ms (Kawasaki et al., 2001). Attention and eye gaze can also be involuntarily drawn towards the expression of emotions within 160–250 ms of being exposed to facial stimuli (Wronka & Walentowska, 2011). Micro-expressions, which are involuntary facial movements that communicate an emotional state within a rapid period of time, can also last as briefly as 250 ms (Yan et al., 2014). Facial micro-expressions of emotional states can occur and inform non-verbal interactions within various community settings. For example, within University settings, it has been observed that undergraduate students can communicate micro-expressions of being sad when viewing scenes of a film where actors are portraying the emotional state of sadness (Grobova et al., 2017). It has also been posited that the recognition of micro-expressions can be integral for health care professionals in their interactions with and assessments of patients who may be attempting to conceal true emotional states (Ekman & Yamey, 2004). Therefore, it is of interest to ascertain if significant differences in emotion recognition, between viewing masked and unmasked faces, are still observed when presented with facial stimuli for a brief 250 ms period.

Social factors that are associated with particular type of facial masks have also previously been seen to influence facial emotion recognition, which suggests that the type of face covering can impede emotion recognition. For example, Kret and De Gelder (2012) observed that Caucasian participants assign lower levels of happiness to faces obscured by a niqab, occluding the nose and mouth area, than faces presented in a fleece cap and knitted scarf where only the eye region was visible. This would suggest that social factors may have influenced how participants perceived the emotions that were being facially expressed when viewing faces obscured by a niqab. Thus, people may assign emotions to others based on social biases attached to particular face coverings. With this in mind, and in the context of the COVID-19 pandemic, people who have engaged with the communal use of face masks have been associated with prosocial attributes such as being altruistic (Cheng et al., 2020; Garber & Vinetz, 2021). In particular, the use of face masks have been associated with higher levels of altruism in frontline health care staff who voluntarily wore facial coverings as means to protect others from the spread of SARS-CoV-2 (Asri et al., 2021). Empathy towards others who are vulnerable to the symptoms of COVID-19 has also been associated with the communal use of face masks (Pfattheicher et al., 2020). However, it is unclear as to whether the process of simply viewing others wearing face masks, during the time of the COVID-19 pandemic, can elicit or encourage particular characteristics such as being empathic towards others.

Empathy has been acknowledged as an important construct in facilitating the rapid recognition of facial emotional states (Martin et al., 1996). Empathy is a construct that refers to the capacity to recognise and mutually share the emotional states as experienced by others (Bonini & Ferrari, 2011; Lieberman, 2007). Atkinson (2007) coined the term *perceptually mediated empathy* to describe the interconnection between the bottom-up processing of facial expressions and the top-down processes of recalling symbolic emotion knowledge. Bottom-up processes refers to the processing of incoming facial stimuli. Whereas top-down processing of facial stimuli refers to the process of utilising schemas or prior knowledge to interpret and facially recognise emotions in others (McRae et al., 2012). The processing of face stimuli has been posited as enabling humans to match the geometric characteristics of facial expressions with emotional schemas. Empathic concern has been identified as a particular facet of empathy that has been associated with the process of facial emotion recognition. Empathic concern has been defined as the process of experiencing compassion and concern for others who appear to be in distress (Stocks et al., 2009). People who score highly on empathic concern, as measured using the Interpersonal Reactivity Index (Davis, 1983), have shown to have greater success rates in recognising negative emotional states, such as anger, fear, sadness, and disgust, when viewing unmasked faces (Israelashvili et al., 2020). Higher levels of self-reported empathy have also been positively associated with the accuracy of facially recognising disgust in others during lab-based scenarios of being exposed to unpleasant smells that elicit the “disgust face” of wrinkled nose and squinted eyes (Fischer et al., 2012). However, it is unclear as to whether there are any associations between levels of empathic concern and emotion recognition when viewing faces that are obscured by facial masks. Thus, it is of interest to ascertain how viewing masked faces may impact levels of empathic concern and capacity to facially recognise the emotional states of others.

It must be acknowledged that at the time of data collection for this study, the community use of face masks within indoor settings, such as public transport and shops, was deemed an essential requirement to mitigate the spread of SARS-Cov-2 and ensure public health (World Health Organisation, 2020). With the necessary and increased use of face masks in public settings, there is a need to ascertain how covering facial features may impact interpersonal communication (Campagne, 2021). This study aimed to use an experimental design to assess how brief observations of faces in opaque facial masks could influence the recognition of all six emotional states as stipulated in Ekman’s (2004) Discrete Emotion Assumption. As utilised by Grundmann et al. (2021), this study used an independent groups’ design to assess the accuracy of emotion recognition between participants who viewed masked faces and those who were presented with unmasked facial stimuli. Furthermore, this study also aimed to ascertain if there were any associations between accuracy in emotion recognition and empathic concern. Therefore, the first hypothesis for this study was that observing faces in masks would significantly reduce emotion recognition for the emotional states of anger, disgust, fear, happiness, sadness, and surprised. This study also aimed to extend the findings of Israelashvili et al. (2020) who observed that people who score highly on empathic concern exhibit greater success rates in facially recognising the emotional states of anger, disgust, fear, and sadness. Thus, this study aimed to investigate if observing masked facial stimuli would elicit higher levels of empathic concern than viewing unmasked faces. Therefore, the second hypothesis was exploratory and posited that participants who viewed masked facial stimuli would self-report higher levels of empathic concern than those who view unmasked faces. Furthermore, it was also an aim to ascertain if higher levels of empathic concern were associated with greater recognition of anger, disgust, fear, and sadness when viewing masked facial stimuli. Thus, the third hypothesis was that positive associations between emotion recognition and empathic concern would be observed when viewing masked facial stimuli.

## Method

### Participants

An a priori G*power analysis indicated that *N* = 196 participants (*n* = 98 per group masked/unmasked) would ensure 80% power in accordance with an alpha level of .05 and medium effect size, *f*^2^(*V*) = 0.0625, (Faul et al., 2009). The inclusion criteria consisted of adults aged 18 years or over who resided in the United Kingdom at the time of data collection. The exclusion criteria composed of any individuals with impaired vision, although participants who wore contact lenses or glasses to correct their vision were eligible to take part in this study.

Recruitment adverts were posted on the social media site of LinkedIn and participants were also recruited via the online platform of Surveyswap https://surveyswap.io/, which enables mutual participation in approved research. Recruitment of participants commenced on 16 December 2020 and ceased 26 January 2021, which was a period of time where the community use of face masks was mandated in the United Kingdom within indoor settings, such as shops and public transport, unless medically exempt. The communal use of face masks in outdoor spaces, where physical distancing could be adhered to, was not mandated in the United Kingdom during the stipulated time period of data collection. The study was conducted online and programmed using Qualtrics. Initially, 244 eligible participants were recorded as having accessed the online link to the study. However, data from 39 participants were removed due to incomplete responses, which is reflective of attrition rates for online studies (Reips, 2002). Thus, this study comprised 199 participants (mean age = 37.44, *SD* = 6.36, range 18–73) who fully completed their participation in the study. Of the 102 participants who were randomly allocated to the condition where all facial stimuli were presented in a mask, *n* = 39 were male (mean age = 43.51, *SD* = 16.90, range 20–69) and *n* = 63 were female (mean age = 32.10, *SD* = 13.93, range 19–68). Of the 97 participants who were randomly allocated to the condition where all facial stimuli were presented with no mask, *n* = 39 were male (mean age = 43.72, *SD* = 16.78, range 20–66) and *n* = 58 were female (mean age = 34.93, *SD* = 15.83, range 18–73).

### Materials

#### The Radboud database of facial stimuli

The model stimuli were obtained from Langner et al.’s (2010) Radboud Faces Database which originally contained frontal static faces of 57 adult models who facially expressed each of Ekman’s (2004) six key emotions: anger, disgust, happiness, fear, sadness, and surprised. Twelve of these models from the database were used (4 female Caucasians, aged ~18–40; 4 male Caucasians, aged 20–45 and 4 male Moroccans, aged 20–50). Thus, 72 images were utilised in this study, in which 12 models facially expressed each of the six emotional states. One hundred and forty-four uncropped images were used in total (72x masked faces and 72 unmasked faces).

**Figure 1.**
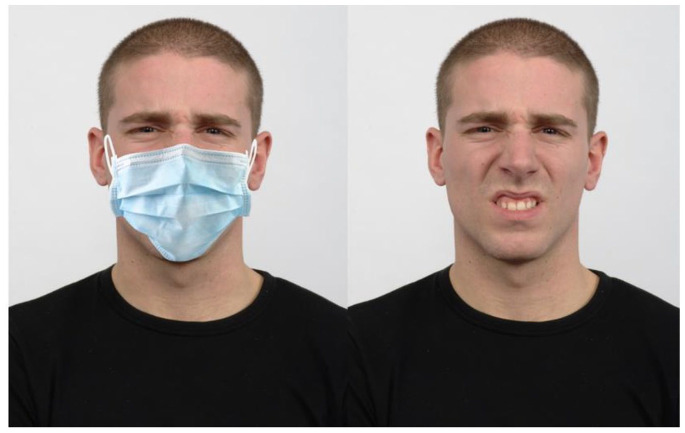
Example stimuli (left masked, right unmasked), with both images of a model presenting the emotion of disgust.

The Radboud Faces Database package has previously been used to investigate emotion recognition (Atkinson & Smithson, 2020). Langner et al.’s (2010) study also validated the facial stimuli to ensure that facial expressions of emotion were of average intensity. As part of Langner’s validation study of the Radboud Database, participants were asked to rate each face, on a five-point Likert-type scale ranging from weak to strong, regarding the intensity at which each emotion was facially expressed. As emotions tend to be facially expressed with average intensity within typical everyday interactions (Motley & Camden, 1988), this study utilised face stimuli where emotions were deemed to be expressed with average intensity as validated by Langner et al. This study also utilised 12 models to present emotions acceptably within one standard deviation of the intensity judgements from all 57 models, while also having a high emotion agreement, in accordance with Langner’s validation of the Radboud Faces Database. See Figure 1 for an example of a model expressing disgust both with and without a mask overlay.

#### Surgical mask overlay

The surgical mask image was obtained from the website named *Free Image* (Surgical Mask Images—Free Vectors, Stock Photos & PSD [freepik.com]), was blue in colour and free to use. The overlay process was conducted via the *Image Online* website (overlay images online [no upload]—Free tool [imageonline.co]). The placement criteria were for the mask ear-loop tops to touch the V between the ear and the head, where the bottom of mask bottom was required to cover the tip of the chin.

#### Static screen

The image named “Silver Glints”, sourced from https://unsplash.com/photos/_EzTds6Fo44?utm_source=unsplash&utm_medium=referral&utm_content=creditShareLink-, was utilised as an afterburn static screen and was free to use with acknowledgement.

#### Video tools with stimuli creation process

The facial stimuli and the afterburn static screen were uploaded onto *individual* MP4 videos. Then, via the website EZGIF (https://ezgif.com/video-to-gif), the videos were then converted to Graphic Interchange Format to allow for a seamless video-to-question-to-video transferral in the Qualtrics survey (integrated with Skip Logic). Thus, each MP4 video, upon uploading to the EZGIF website, was initially scaled to project at an automatic pixel height X 480 pixels wide (or maximum width of phone screen) at 10 frames per second via the FFMPEG method. The converted GIF was then o*ptimised* by a compression level of 35, which minimised the file size to increase the desired Qualtrics performance potential—but to still keep a satisfactory amount of image quality.

#### Interpersonal Reactivity Index

Empathic concern was assessed using a seven-item subscale within the Interpersonal Reactivity Index (IRI) (Davis, 1983). This seven-item subscale requires participants to respond on a four-point Likert-type style ranging from 1 = *Does not describe me well* to 4 = *Describes me well*, with higher scores indicating greater levels of empathic concern. Example items within the empathic concern subscale are *I am often quite touched by things that I see happen, I often have tender, concerned feelings for people less fortunate than me, and When I see someone being taken advantage of, I feel kind of protective towards them.* The seven-item subscale, within the IRI, has previously been used to measure empathic concern across the lifespan (O’Brien et al., 2013), within community volunteers who engage in post-disaster relief work (Cristea et al., 2014) and in association with human interactions with artificial intelligence (Darling et al., 2015). The Cronbach’s alpha for the empathic concern subscale is reported as having good internal consistency, *α* = .80.

#### Procedure

**Figure 2.**
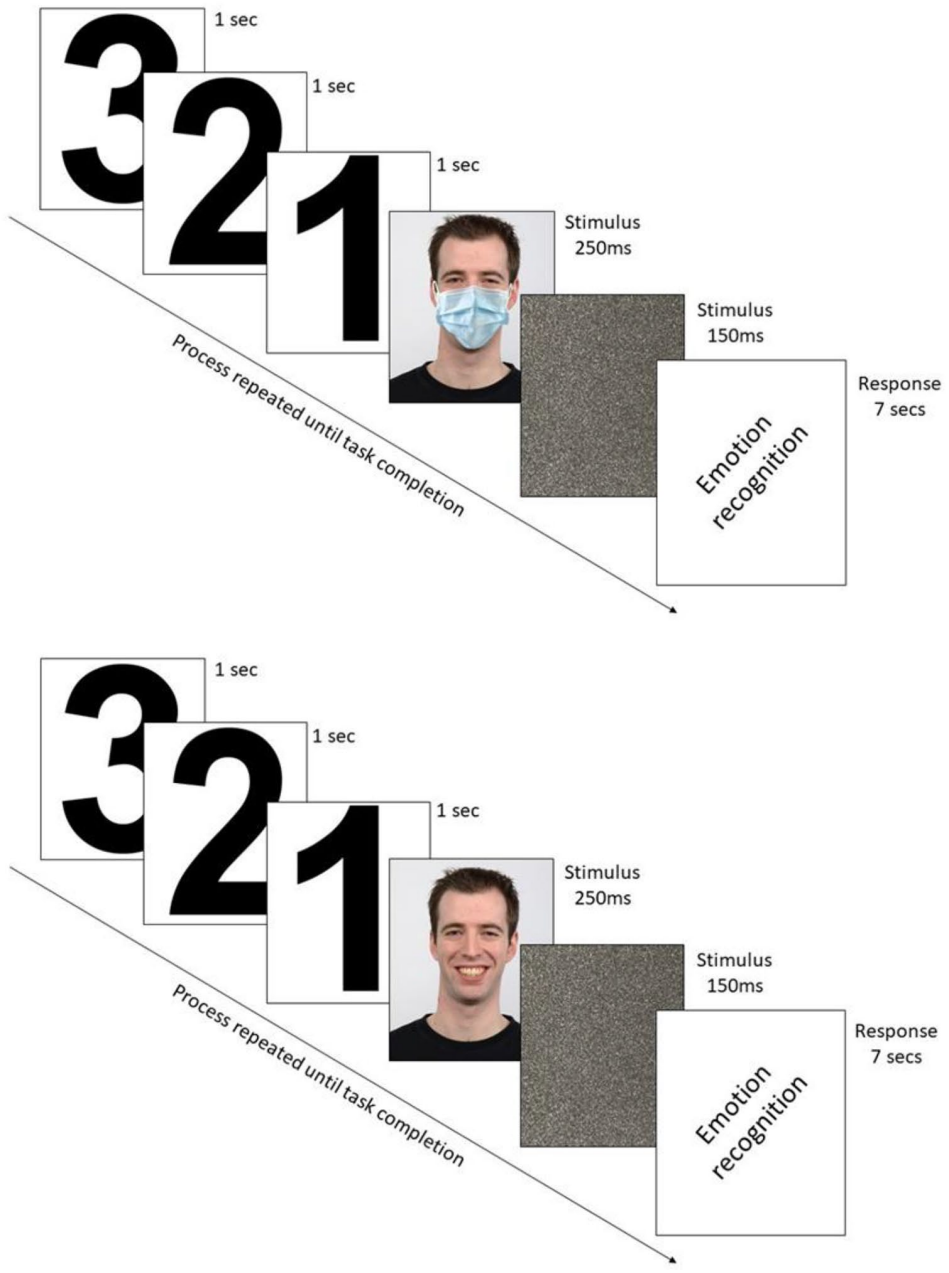
After a 3-s countdown to opaque masked/unmasked faces, the respective groups indicated the posed emotion (presented for 250 ms) after selecting from six possible emotion options (Happy, Sad, Fear, Anger, Surprise, or Disgust) within 7 s.

This study obtained ethical approval from the Ethics Committee at the School of Health and Life Sciences, University of Northumbria at Newcastle (Ethics Committee REF: 26628). Participants were provided with the link to the Qualtrics platform and were asked to complete the study within a quiet and secluded room. As a means to document informed consent to take part in this study, participants were asked to click on an icon to confirm that they agreed to participate. Participants then confirmed their age, gender, and eligibility to take part in this study. After random assignments to either the *masked* or *unmasked* condition, participants then initially faced two *practice* stimuli-videos that both aligned with their appointed groups (either *masked*/*masked* or *unmasked/unmasked*) for the main task. Each video had a visual 3-s countdown before the facial stimuli was presented for 250 ms only then followed by a 150 ms afterburn static screen. The images of facial stimuli were presented for a period of 250 ms, as it has been observed that visual attention to facial expression of emotions can occur within 160–250 ms of being presented with an image of a face (Wronka & Walentowska, 2011). After each 250 ms presentation of facial stimuli, participants were automatically directed to answer what type of emotion was being facially expressed. Participants were presented with six possible response options (*Happiness*, *Sadness*, *Anger*, *Disgust*, *Surprised*, *Fear*; in that order). Providing participants with a 7-s response time, when viewing a battery of facial stimuli has previously been used to assess facial emotion recognition in non-clinical populations (Seo et al., 2020). Thus, participants had 7 s to provide their response before being directed to the next visual image of a face (or *instantly* once the response had been pressed, see Figure 2).

Upon completion of the practice rounds, participants were then presented with another screen detailing that they would be presented with 36 images of faces, followed by a 1-min rest period, and then recommence with being presented with a further 36 images of faces. Upon pressing go, participants then replicated the same process as the practice round, within their assigned condition, for 36 presentations of facial images. The presentation of each face lasted 250 ms. After each presentation of a face, participants were asked to state which of the six emotions was being facially expressed. After the 36th presentation of the facial images, participants were provided with a 1-min rest period. Following the 1-min rest, the page automatically directed participants to the next 36 images of faces. Again, each face was presented for 250 ms. After each face had been presented, participants were asked to state which of the six emotional states had been facially expressed by the model. Participants then completed the seven-item subscale, within the IRI, that measured empathic concern. Finally, participants were guided to a debrief page. The average time that it took participants to complete their participation in this study was 15 min.

## Results

### Emotion recognition

A two-way mixed ANOVA was conducted to assess if viewing masked faces would elicit reductions in the capacity to facially recognise emotional states. The between groups factor of “facial covering” had two levels in which participants were randomly allocated to viewing facial stimuli that were presented as either 1) masked or 2) unmasked. The repeated measures factor was the “type of facially expressed emotions,” which had six levels: 1) *anger, 2) disgust, 3) fear, 4) happiness, 5) sadness*, and *6) surprised.* The dependent variable was the success rate of facial emotion recognition. Emotion recognition scores, for each of the six individual emotional states, could range from 0 to 12. Therefore, total emotion recognition scores ranged from 0 to 72. The dataset for this study can be found at the following link: https://osf.io/sc65v/

A significant main effect of “facial covering” indicated that emotion recognition was significantly lower when viewing masked faces (*M* = 40.33, *SD* = 6.73) in comparison to participants who viewed unmasked facial stimuli (*M* = 56.22, *SD* = 7.20), *F*(1,197) = 245.06, *p* < .001, *ηp* ^2^ = .55).

There was also a significant main effect of “type of facially expressed emotion” on facial emotion recognition accuracy, *F*(5,985) = 260.59, *p* < .001, *ηp* ^2^ = .57. With a Bonferroni corrected alpha (α = 0.008), participants were observed to be most successful in recognising the facial expression of *happiness* (*M* = 11.05, *SD* = 1.60). There was a greater level of accuracy in facially recognising the emotion of *happiness* than *surprised* (*M* = 10.08, *SD* = 1.94, *p* < .001). There was then greater success in the facial recognition of *surprised* in comparison to *sadness* (*M* = 8.36, *SD* = 2.73, *p* < .001). There was greater successful recognition of *sadness* in comparison to *anger* (*M* = 7.39, *SD* = 2.37, *p* < .001). However, there was no significant difference between the facial recognition of *anger* and *disgust* (*M* = 6.67, *SD* = 3.38, *p* = .038). Although there was greater success in the facial identification of *disgust* in comparison to *fear* (*M* = 4.73, *SD* = 3.32, *p* < .001).

There was also a significant interaction effect between “facial covering” and “type of facially expressed emotion,” *F* (5,985) = 35.90, *p* < .001, *ηp* ^2^ = .15. To interpret this interaction, simple main effects analyses were conducted using a Bonferroni corrected α of 0.008.

**Figure 3.**
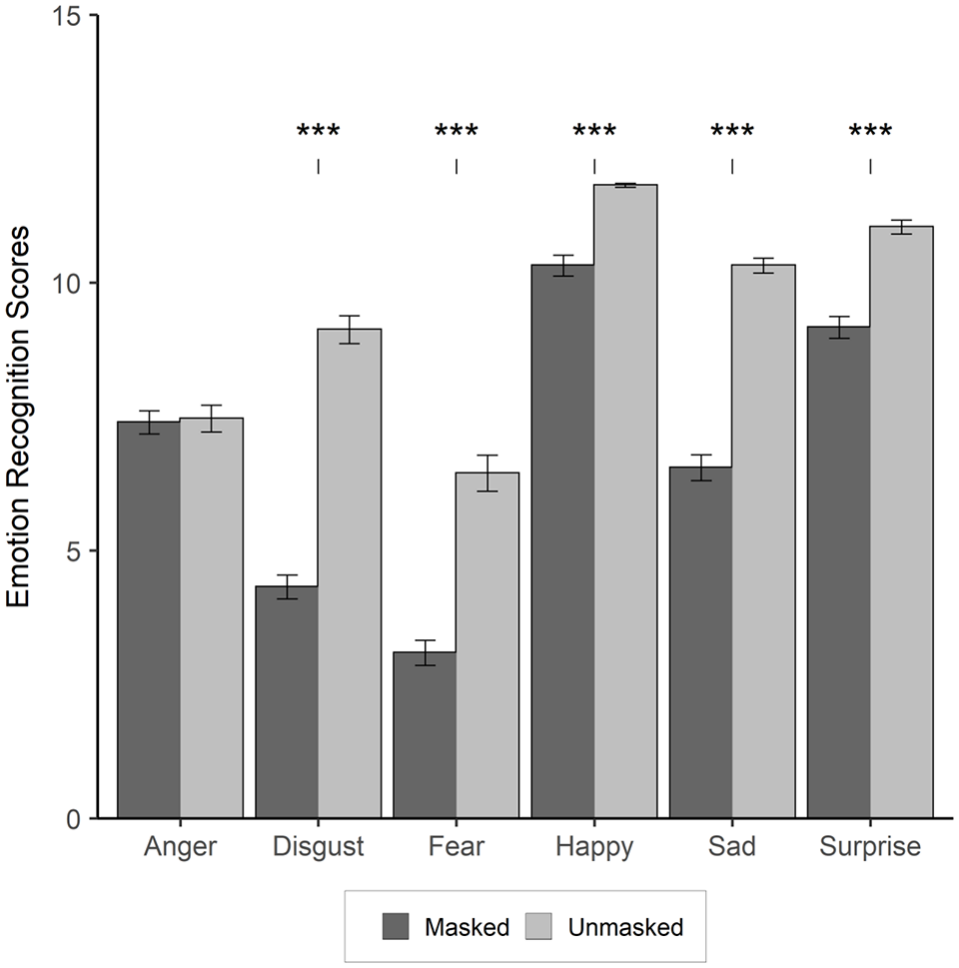
An illustration of the total correct facial emotion recognition scores between the masked and unmasked conditions for the emotional expressions of *anger, fear, sadness, surprised, happiness*, and *disgust* standard error bars (Note *** = *p* < .001).

There was a simple main effect of “facial covering” in which viewing masked faces significantly reduced recognition of *happiness*, *t*(197) = 7.60, *p* < .001; *d* = 1.09, *sadness*, *t*(197) = 13.80, *p* < .001; *d* = 1.96, *fear*, *t*(197) = 8.20, *p* < .001; *d* = 1.16, *surprised*, *t*(197) = 7.83, *p* < .001; *d* = 1.11, and *disgust*, *t*(197) = 14.18, *p* < .001; *d* = 2.01. However, there was no simple main effect of “facial covering” on faces presenting with *anger*, *t*(197) = .447, *p* = .66; *d* = 0.06. See Table 1 and Figure 3 for illustrations of the descriptive statistics.

**Table 1.** The means (standard deviations) of the total correct emotional interpretations towards disgust, anger, happy, surprise, sad, and fear for the masked and unmasked conditions (*n* = 97 unmasked, *n* = 102 masked).

	Unmasked	Masked
Disgust	9.12 (2.55)	4.33 (2.21)
Anger	7.46 (2.54)	7.31 (2.20)
Happiness	11.82 (0.38)	10.30 (1.93)
Surprise	11.04 (1.31)	9.16 (2.00)
Sadness	10.32 (1.39)	6.50 (2.37)
Fear	6.44 (3.33)	3.10 (2.36)

As Grundmann et al. (2021) and Ruffman et al. (2008) have observed that age can influence facial emotion recognition, an Analysis of Covariance was conducted with age as the covariate. After the Homogeneity of Regression slopes assumption was met via non-significant interactions between the independent groups factor of facial covering and the covariate (age), *F* (1, 195) = .257, *p* = .613, it was discovered that viewing masked faces still significantly reduced the overall accuracy in facial emotion recognition, *F* (1, 196) = 309.30, *p* < .001. Post-hoc comparisons with adjusted means found participants viewing unmasked faces had significantly (*p* < .001) greater accuracy in facial emotion recognition (*M* = 56.38, *SE* = 0.66) in comparison to observing masked facial stimuli (*M* = 40.18, *SE* = 0.64).

To investigate how emotion recognition was more or less impacted by observing masked and unmasked faces, two Freidman tests were performed on the emotion recognition scores across both conditions. The analyses found a significant effect of emotion in the unmasked, χ^2^(5,97) = 286.43, *p* < .001, *W* = .591, and masked, χ^2^(5,102) = 344.74, *p* < .001, *W* = .676, conditions, indicating there was a difference in the success levels that participants recognised each individual emotion within each condition. Please refer to Table 2 for an illustration of the success rate at which emotional state were recognised within the masked and unmasked conditions.

**Table 2.** Confusion matrices illustrating the expressed emotions of facial stimuli and responses of participants (*n* = 97 unmasked, *n* = 102 masked).

Unmasked faces						
Expressed emotion						
Perceived emotion	Fear	Surprise	Disgust	Anger	Sad	Happy
Happiness	0.34%	0.86%	0.60%	0.59%	0.85%	**98.45%**
Sadness	28.60%	0.42%	0.93%	15.18%	**86.01%**	0.17%
Anger	10.13%	0.51%	18.47%	**57.98%**	5.75%	0.08%
Disgust	2.76%	1.29%	**75.93%**	16.93%	5.15%	0.34%
Surprise	3.79%	**91.93%**	2.24%	5.23%	0.77%	0.51%
Fear	**53.69%**	4.29%	1.45%	2.77%	0.60%	0.08%
No response	0.68%	0.69%	0.33%	1.12%	0.87%	0.34%
Masked faces						
Expressed emotion						
Perceived emotion	Fear	Surprise	Disgust	Anger	Sad	Happy
Happiness	1.16%	2.21%	8.34%	1.30%	2.71%	**85.86%**
Sad	2.61%	7.12%	9.55%	9.07%	**54.32%**	4.00%
Anger	2.55%	1.57%	39.63%	**61.03%**	10.88%	2.69%
Disgust	4.66%	4.24%	**36.13%**	21.17%	14.71%	3.28%
Surprise	62.42%	**76.40%**	2.71%	2.71%	4.50%	2.63%
Fear	**25.82%**	7.44%	3.36%	4.24%	12.08%	0.90%
No response	0.83%	1.08%	0.33%	0.50%	0.83%	0.67%

In the masked condition, happiness was identified with the greatest level of accuracy in comparison to surprised, *t*(202) = 4.16, *p* < .001. Surprised was then recognised with greater accuracy than anger, *t*(202) = 6.26, *p* < .001, followed by sadness, *t*(202) = 2.54, *p* = .02, then disgust, *t*(202) = 6.76, *p* < .001. Participants had the least accuracy of identifying the emotion of fear within the masked condition, *t*(202) = 3.86, *p* < .001.

When viewing unmasked faces, participants again had the greatest accuracy in recognising the emotion of happiness, which was significantly greater than surprised, *t*(192) = 5.67, *p* < .001, followed by sadness, *t*(192) = 3.73, *p* < .001, then disgust, *t*(192) = 4.06, *p* < .001, then anger, *t*(192) = 4.54, *p* < .001, and with fear being the emotional expression being identified with the least amount of accuracy, *t*(192) = 2.40, *p* = .017.

To understand more closely how particular emotional states can be misidentified as another emotion, a confusion matrix was conducted for both the unmasked and masked conditions (see Table 2). This matrix clearly showed participants viewing masked faces had lower corroborations between the expressed emotion and their perceived emotion. Most strikingly, the expressed emotion of fear was incorrectly misidentified as surprised on 62.42% of occasions within the masked condition. The emotion of disgust was misidentified as anger in 39.63% of occasions in the masked condition. The facial expression of anger was also misidentified as disgust on 21.17% of occasions within the masked condition. This would suggest that the facial expressions of fear, anger, and disgust may be most susceptible to inaccuracies in emotion recognition when observing masked faces within a brief period of time.

A comparison of each emotion’s (happiness, sadness, anger, disgust, surprise, and fear) observed responses against the corresponding expected values, using chi-square tests, was conducted to analyse the direction of errors in facial emotion recognition. The emotions with observed values that significantly exceeded the average chi-square’s expected values for that particular emotion category (correct/error) were as follows. In the unmasked condition, the emotion of fear was frequently mistaken for the emotion of surprised, χ^2^ (1, 97) = 1899.89, *p* < .001, and disgust, χ^2^ (1, 97) = 124.64, *p* < .001. The emotion of disgust was regularly misinterpreted as anger, χ^2^ (1, 97) = 665.25, *p* < .001, and the emotion anger was often misidentified as sadness, χ^2^ (1, 97) = 402.48, *p* < .001, and disgust, χ^2^ (1, 97) = 532.59, *p* < .001.

In the masked condition, the emotion of fear was misinterpreted as surprise twice as often, χ^2^ (1, 102) = 5151.52, *p* < .001. The emotion of disgust was misinterpreted more often as anger, χ^2^ (1, 102) = 1774.67, *p* < .001. Anger was regularly misinterpreted as disgust, χ^2^ (1, 102) = 328.02, *p* < .001. Finally, sadness was frequently misinterpreted as disgust, χ^2^ (1, 102) = 94.41, *p* < .001.

### Empathic concern

A between subjects *t*-test was conducted to ascertain if there were differences in the levels of self-reported empathic concern between participants who viewed facial stimuli in masks compared with the group who viewed unmasked faces. The results indicated that participants who viewed masked faces reported higher levels of empathic concern (*M* = 23.35, *SD* = 3.44) than participants who were required to view unmasked faces (*M* = 22.42, *SD* = 3.22), *t*(197) = 1.97, *p* = .05; *d* = 0.28.

Further analysis was conducted to assess if there was any association between levels of empathic concern and accuracy of facial emotion recognition when participants viewed masked facial stimuli. A bivariate correlation revealed that higher levels of empathic concern was significantly associated with greater accuracy in overall emotion recognition when viewing masked faces, *r* = .23, *p* = .02, although this failed to reach significance in the unmasked condition *r* = .09., *p* = .40. Further bivariate correlations were conducted to assess the relationships between empathic concern and recognition of anger, disgust, fear, happiness, sadness, and surprised when viewing masked faces. A positive association was observed between levels of empathic concern and success rate of recognising the emotional state of disgust when viewing masked faces, *r* = .20, *p* = .04. However, when viewing masked faces, there were no significant associations between empathic concern and facial recognition of anger (*r* = .12, *p* = .22), fear (*r* = .03, *p* = .78) happiness (*r* = .16, *p* = .12), sadness (*r* = .14, *p* = .16) and surprised (*r* = .07, *p* = .49).

## Discussion

This study investigated how viewing masked faces for a brief period of time could impact emotion recognition and empathic concern. First, it was hypothesised that brief exposure to masked faces would significantly reduce facial emotion recognition. The results indicated that viewing faces, covered by opaque masks, significantly reduced the ability to facially recognise the emotional states of happiness, sadness, disgust, fear, and surprised. However, there was no significant difference in emotion recognition accuracy between viewing masked and unmasked faces that expressed anger. This finding extends the results observed by Carbon (2020) and Grundmann et al. (2021) who also found that viewing masked faces can significantly reduce emotion recognition when observing facial stimuli for an unlimited or 2-s exposure period, respectively. The observation that significant reductions in emotion recognition can occur when viewing masked faces for a brief period of 250 ms has some important implications when considering how facial micro-expressions can inform and help facilitate cohesive social interactions.

As public health initiatives have encouraged the increased global use of facial coverings to reduce the transmission of SARS-CoV-2 (World Health Organisation, 2020), this has led to an increased use of face masks in community settings. With the results observed in this study, it could be argued that any non-verbal interactions in community settings, that comprise of brief exposure to masked faces, could inhibit the expression and recognition of emotional states. It has been noted that humans can facially recognise the emotional states of others within 120–170 ms (Kawasaki et al., 2001) and that facial expressions can be an important method to communicate emotional states in a non-verbal manner (Phutela, 2015). It has also been recognised that the detection of facial micro-expressions of emotions, which last as briefly as within 250 ms, can be important in facilitating frontline health care staff to facially recognise the emotional states of their patients (Ekman & Yamey, 2004). However, this study would suggest that face masks can block areas of the face that are essential for the non-verbal communication of emotional states during brief interactions. When masks inhibit effective communication, this can potentially lead to stressful and incoherent social interactions within community settings (Campagne, 2021). It is, therefore, necessary for future research to investigate if there is a relationship between emotion recognition and state aspects of psychological wellbeing during brief social interactions in which facial coverings are worn. It is also essential to further investigate how the communal use of face masks can impact interactions in settings, such as health care, where the facial recognition of emotional states can be integral in ensuring the welfare of others who may be experiencing negative emotions, such as sadness.

This study also assessed how viewing masked faces could impact the facial recognition of specific emotional states. First, it was observed that the highest success rate in emotion recognition, across both the masked and unmasked conditions, was for the emotional state of happiness. Participants successfully recognised the emotional state of happiness on 98.5% of occasions in the unmasked condition and 85.8% of occasions in the masked condition. This would suggest that the eye region of the face can still convey happiness to facilitate a high success of emotion recognition when viewing masked facial stimuli. It should also be noted that the success rates of facially recognising happiness tends to be higher than the recognition of other emotions, even when the face is inverted (Kirita & Endo, 1995), blurred (Endo et al., 1995) or in the periphery (Calvo et al., 2014).

This study also observed that the greatest decline in emotion recognition occurred when viewing masked faces that were expressing the emotional state of disgust; with a 76% success rate of recognising disgust when viewing unmasked faces decreasing to a 36.1 % success rate when viewing masked faces expressing disgust. It has been posited that facial recognition of disgust typically relies on observing the lower portion of the face (Blais et al., 2012; Wegrzyn et al., 2017). Thus, the communication and recognition of disgust could be impaired significantly by opaque facial coverings that block the nose and mouth areas of the face.

This study revealed that when participants viewed masked faces, the facial expression of fear was misidentified as surprised on 62.42% of occasions. It was previously observed by Carbon (2020) that emotion recognition for fearful faces was not significantly impaired when viewing masked facial stimuli. However, it must be noted that the battery of facial stimuli used by Carbon (2020) did not comprise models who facially expressed the emotional state of surprised. This study used the Radboud Faces Database, which includes facial stimuli that express both fear and surprised. Thus, utilising a database of faces that included the facial expression of surprised could explain why fear was identified with the least accuracy when viewing masked faces. Fearful and surprised face expressions involve similar facial movements that comprise widening the eyes and opening of the mouth (Duan et al., 2010; Schroeder et al., 2004). The findings of this study would suggest that occlusion of the mouth area and observing facial stimuli in which eyes appear to have widened, could lead to misidentifying fear as the emotional state of surprised when viewing masked faces. However, fear and surprised have different emotional meanings. Fear has been defined as an emotional reaction to a perceived threat of physical or psychological harm (Adolphs, 2013). While surprised has been defined as the emotional reaction to an unexpected event that could be evaluated either positively with joy or negatively (Derbaix & Vanhamme, 2003). The results of this study would suggest that when viewing masked faces, people may be susceptible in assigning the emotional state of surprised to people who are experiencing fear and a perceived threat. This has important implications as the ability to facially recognise fear in others can prompt observers to engage in prosocial or helpful behaviours (Marsh & Ambady, 2007). The notion that facial expressions of emotions can influence behavioural responses is also illustrated in Van Kleef’s (2009) Emotion as Social Information Model (EASI). The EASI model illustrates how people may respond to or react in accordance with the emotions that they identify in others through the non-verbal recognition of facially expressed emotions. Thus, the inability to recognise the emotional state of fear, when observing masked faces during brief interactions, may prevent people from responding and assisting others accordingly. However, it must be acknowledged that fear was also facially recognised with the least level of accuracy in the unmasked condition in this study. Previous studies that have used the Radboud Faces Database have reported that the facial expression of fear can be confused with surprised by school aged children (Verpaalen et al., 2019) and adults (Langner et al., 2010), which converges with this study. Thus, it could be that the facial expressions of fear and surprised within the Radboud Faces Database can be perceived as being similar, which may explain why fear was facially recognised with the least level of accuracy in both the masked and unmasked conditions in this study.

This study also observed that viewing masked faces could significantly reduce recognition of the facial expressions of happiness, sadness, surprised, and disgust. However, interestingly, there was no significant difference in the facial recognition of anger between viewing masked and unmasked faces. This could be explained by an evolutionary perspective that facial recognition of anger is particularly important in ensuring survival and evading perceived threats from others (van Honk & Schutter, 2007). It has been argued that humans are able to detect facial emotional displays of threat, such as anger, much more efficiently than non-threatening emotions such as happiness (Eastwood et al., 2001). It has been posited that the downwards pointing of eyebrows associated with the expression of anger could be the facial cue that elicits efficient detection of angry facial expressions and potential threat (LoBue & Larson, 2010). In this study, it could be that participants were able to detect facial expressions of anger with the same level of accuracy, regardless of viewing masked or unmasked faces, as the facial masks did not occlude the eye or brow area.

It was also hypothesised that higher levels of empathic concern would be associated with greater emotion recognitions. A positive association was observed between empathic concern and overall accuracy in emotion recognition when viewing masked faces. Furthermore, higher levels of empathic concern were associated with the facial emotion recognition of disgust when viewing masked faces. This finding extends the previously observed associations between self-reported empathy and accuracy of facially recognising disgust when observing unmasked faces (Fischer et al., 2012). Disgust has been defined as the emotional response to facilitate the withdrawal from threats that may cause bodily contamination and harm (Haidt et al., 1997). From an evolutionary perspective, the emotion of disgust has evolved as a means to protect humans from consuming or being exposed to harmful pathogens and toxic substances (Rozin et al., 2008). It has been observed that being presented with stimuli that causes concerns of being contaminated, such as observing someone sneezing on food, can elicit the “disgust face” that comprises a wrinkled nose, squinted eyes and gaping mouth (Stevenson et al., 2010). It has previously been acknowledged that people with higher levels of empathic concern may be more sensitive in recognising when other people are experiencing distress (Stocks et al., 2009) and negative emotional states, such as disgust (Israelashvili et al., 2020). It could be that even when viewing masked faces for a brief period of time, people with higher levels of empathic concern may have the sensitivity required to recognise the emotional state of disgust and when others perceive a risk of contamination. It is also necessary to consider the EASI model (Van Kleef, 2009) and how facial cues can encourage behavioural responses of observers. It has been recognised that experiencing disgust can elicit withdrawal related behaviours towards sources that may present as risks of being contaminated (Rozin, 2015). Thus, it would be of interest to further investigate if empathic concern is a characteristic that may help to facially recognise disgust when observing masked faces and if this process also elicits withdrawal related behaviours. Therefore, it is recommended that further research is conducted to further investigate how trait and state levels of empathic concern may affect the capacity to facially recognise potential distress or disgust as experienced by others during interactions that involve the application of face masks.

This study also observed that participants, who were assigned to the condition of viewing masked facial stimuli, self-reported higher levels of empathic concern than those who viewed unmasked faces. The effect of face masks on perceived empathy has received some notable attention. For example, it has been argued that health care professionals who wear face masks can be perceived as less empathic than those who do not wear facial coverings (Wong et al., 2013). However, the findings of this study would suggest that the process of viewing masked faces may elicit greater empathic concern within the observer. This converges with Asri et al. (2021) and (Wollslager, 2021) who observed that ~80% of their participants viewed that wearing face masks is a demonstration of showing concern for the health of others. This is an interesting finding given that the communal use of face masks was mandated in the United Kingdom, as a public health strategy to mitigate the spread of SARS-CoV-2, at the time of data collection. It has been argued that the process of providing details on how the use of face masks can help protect people with immune disease, and who are at high risk of the symptoms associated with SARS-CoV-2, can potentially elicit state empathy and adherence to public health strategies to prevent the spread of COVID-19 (Pfattheicher et al., 2020). In this study, it is unclear as to whether the process of viewing masked facial stimuli alone was the main cause of eliciting greater empathic concern than when viewing unmasked faces, given the public health campaign that coincided with the data collection. It could be that the process of attempting to facially recognise emotions, when faces are obscured by masks, could also elicit greater empathic concern. It is therefore necessary to conduct further research to ascertain how the interaction between viewing masked/ unmasked faces and capacity for facial emotion recognition can impact empathic concern within the observer.

There are some limitations that need to be considered when interpreting the results of this study. First, the static facial stimuli used in this study may not fully represent real-life fluid interactions that occur within community settings (Trautmann et al., 2009). More specifically, previous studies (Carbon, 2020; Grundmann et al., 2021) have used a more sophisticated mask-overlay methodology, with dark shading included to ensure contour realism and stricter ear-loop placements (overlapping the ears) superseded the current mask-overlay placements, which instead failed to cover the whole chin, and had mask ear-loops persisting in-front of the ears. Therefore, future research could incorporate real-life face-to-face interactions to help address the present limitations and to advance the understanding of how face masks can influence emotion recognition. This would help to increase ecological validity and better replicate every-day interactions that people have in communal settings. Furthermore, future studies may also consider utilising eye tracking technologies when assessing how face masks impact emotion recognition. Although visual attention can be involuntarily drawn to facial expressions of emotion (Wronka & Walentowska, 2011), this study did not collect data relating to the eye gaze of participants during presentation of facial stimulus. Thus, eye tracking technologies that monitor eye gaze may help to ascertain the areas of masked faces people visually attend to when attempting to recognise emotions.

It must also be acknowledged that this study did not include neutral facial expressions within the battery of face stimuli that were presented to participants. It has previously been observed that the facial expressions of anger, disgust, happiness, and sadness can be misinterpreted as neutral expressions when viewing masked faces (Carbon, 2020). It has also been observed that observing neutral facial expressions can elicit similar activation of the neural substrates as when viewing faces that are expressing specific emotional states even though the neutral face conveys no explicit emotional message (Carvajal et al., 2013). In accordance with the EASI model (Van Kleef, 2009), it is important to consider how confusing neutral facial expressions with specific emotional states could elicit inappropriate behavioural responses. For example, situations whereby an observer misinterprets anger to someone who has a neutral expression when wearing a mask could elicit an inappropriate behavioural response of either evasion or encouraging an aggressive act from an observer. Given that the misidentification of neutral facial expressions may lead to incongruent social interactions, there is a necessity for subsequent studies to include neutral facial expressions within the battery of stimuli when investigating the impact of face masks on emotion recognition.

It is also important to consider that the utility of an independent groups’ design was used to investigate how masks may influence the accuracy of facial emotion recognition. Although this type of design has previously been included in studies that have investigated this area of research (Grundmann et al., 2021), it must be acknowledged that individual differences within the participants may have influenced the results of this study. For example, this study did not consider how the ethnicity of participants may have influenced the ability to facially recognise emotions when observing masked and unmasked faces. This is an important consideration given that ethnicity has been observed to influence facial emotion recognition (Elfenbein & Ambady, 2002). Therefore, subsequent studies that use independent groups’ design could match participants on variables that have previously been shown to influence accuracy in facial emotion recognition, such as ethnicity (Elfenbein & Ambady, 2002), age (Montagne et al., 2007), and gender (Abbruzzese et al., 2019). Furthermore, repeated measures designs could also be used to negate the influence of individual differences within participants when assessing the influence of masks on facial emotion recognition.

In summary, this study aimed to investigate how brief exposure to masked faces could influence facial emotion recognition. It was also an aim to ascertain if emotion recognition of masked faces would be associated with levels of empathic concern. It was observed that brief observation of masked faces elicited significant reductions in facially recognising the emotional states of disgust, fear, happiness, sadness, and surprised. However, there were no differences between the brief observation of masked and unmasked faces in the success rate of recognising anger. Furthermore, empathic concern was observed to be positively associated with overall emotion recognition and success rate in recognising the emotional state of disgust. It is acknowledged that the use of facial coverings in community settings is integral to public health strategies that aim to reduce transmission of the SARS-CoV-2 virus. Thus, there has been an increase in the number of brief interactions whereby people observe others wearing face masks within essential community settings, such as food stores. The results of this study would suggest that people may have difficulties in facially expressing or recognising emotional states during brief interactions with others. To further facilitate the community use of face masks, it is necessary to ascertain how disruption to emotion recognition may impact state aspects of psychological wellbeing during brief interactions in communal settings. Further research and strategies that support effective non-verbal communication of emotional states, alongside the community use of face masks, could be integral in reinforcing public health strategies that advocate the use of facial coverings to mitigate the transmission of airborne viruses.
